# *ADD1* Single Nucleotide Polymorphisms Are Associated With Essential Hypertension Among Han and Mongolian Population in Inner Mongolia Area

**DOI:** 10.3389/fgene.2022.931803

**Published:** 2022-08-12

**Authors:** Yi Zhang, Peiye Chang, Zhiyue Liu

**Affiliations:** ^1^ Department of Medicine, Luoyang Polytechnic, Luoyang, China; ^2^ Department of Basic Medicine, Inner Mongolia Medical University, Hohhot, China; ^3^ Department of Nuclear Medicine, First Affiliated Hospital of Inner Mongolia Medical University, Hohhot, China

**Keywords:** essential hypertension, ADD-1, CYP11B2, single nucleotide polymorphism, genetic, risk factor

## Abstract

Aldosterone synthase (*CYP11B2*) and α-adducing (*ADD1*) are candidate genes that play key roles during essential hypertension (EH) incidence. However, the association between their genetic mutations and the risk of EH is unclear. The present study investigated specific single nucleotide polymorphisms (SNPs) from *CYP11B2* and *ADD1*, and their potential role as risk factors for EH based on 423 Mongolian and 410 Han people in Inner Mongolia province. In the allelic model, people with *ADD1* rs2239728-A presented a 0.74-fold risk than rs2239728-C, whereas the *ADD1* rs4961-T was associated with a 1.37-fold higher risk than allele G in the Han population. The genetic model reported that the rs2239728-A carrier (AA + AC) was 0.59-fold lower than the CC carrier, whereas the rs4961-G carrier (GG + GT) was 0.59-fold lower than the TT carrier in the dominant model. After gender adjustment, people with rs2239728-A was a 0.63-fold risk than –C in EH, but the rs4961-T carrier was associated with a 1.63-times higher risk than –G in females. Haplotype analysis showed that GCCT was associated with essential hypertension in the Han population, and it was a risk factor for EH. Our identification reported novel SNPs of *ADD1* with protective significance for EH among females in the Chinese Han population, together with its haplotype GCCT as a risk factor for EH.

## Introduction

Essential hypertension (EH) is a common disease that can adversely impact human health conditions and cause a variety of other physical and pathological disorders in the heart, kidney, or brain, such as stroke, myocardial infarction, and renal failure (Lewington et al., 2002; Ju et al., 2003). The age of patients who sustain EH has become younger these years and with an increasing trend. A previous exploration reported that over 972 million people are suffering from EH all over the world, including 270 million patients in China (Chugh et al., 2014), where the total number will approximately increase to one-third of the world population in 2025 (Kearney et al., 2005). Furthermore, EH is a multifactorial disease that is affected by environmental, physiological, and genetic factors as well as their interactions (Shin et al., 2004; Belmonte and Blaxall, 2011; Savoia et al., 2011; Wang et al., 2014). Previous studies have shown that heritage is the major cause of interindividual variability in human blood pressure (BP) (Luft, 2001; Tobin et al., 2005). A population-based assessment made by Tobin’s group further demonstrated the heritability of 24-h human average BP was approximately 65% (10). In addition, Hottenga’s group reported the heritability of human BP is related to genetic polymorphisms with a range of 31–68% (Hottenga et al., 2005). SNPs are more suitable for association researches because of their stable and biallelic feature (Syvänen, 1998). Thus, they have been widely investigated with their variations that happen in a single base pair in the DNA sequence (Chen et al., 2017). Detailed exploration of genetic polymorphisms in EH would be a great help for identifying functional antihypertensive targets and detecting specific risk factors for clinical prediction.

*CYP11B2* and *ADD1* are two candidate genes that play key roles in the water–electrolyte metabolism; the disorder of this biological process is regarded as a vital pathogenic factor of EH. *CYP11B2* encodes a member of the cytochrome P450 superfamily of enzymes. The cytochrome P450 proteins are monooxygenases that catalyze many reactions involved in drug metabolism and synthesis of cholesterol, steroids, and other lipids. More importantly, the synthesis and lysis of aldosterone are accomplished using aldosterone synthase (*CYP11B2*). Aldosterone is the key effector of the rennin angiotensin aldosterone system, which is a significant regulator of vascular content and BP, and also plays a key role in regulating water–electrolyte metabolism, maintaining internal environment stability and vascular tension. Alpha-adducing (*ADD1*) is a vital member of adducins, which is a family of cytoskeletal proteins encoded by three genes (alpha, beta, and gamma). These family members of adducins were reported to participate in various types of cellular signal transportation and membrane–ion transport, including Na + –H+ exchange, Na + –Li + countertransport, and Na + –K + –Cl-cotransport (Tripodi et al., 1996). These biological processes are all major parts of water–electrolyte metabolism, which is highly associated with the risk of EH. However, the relationship between EH risk and genetic polymorphisms of both genes is still undefined (Conen et al., 2009; Niu et al., 2010; Liu et al., 2011; Zhang et al., 2013). We speculated the reason might be the difference in population and area. Inner Mongolia is an area with high morbidity of EH, but exploration on a genetic level to investigate whether mutation of gene sites affects the risk of EH is still lacking. Our study aims to explore the association between genetic polymorphisms of *CYP11B2* and *ADD1* with EH in the Han and Mongolian populations in the Inner Mongolia area.

## Methods and Materials

### Research Individuals

Our present study included 423 Mongolian subjects (208 EH patients, including 139 males and 69 females; 215 healthy controls, including 113 males and 102 females) and 410 Han people (198 EH patients, including 112 males and 86 females; 212 healthy controls, including 121 males and 91 females) from the First Affiliated Hospital of Inner Mongolia Medical University with no limitation of age. All the subjects were living in Inner Mongolia province with genetically unrelated ethnic. We asked every single individual to complete a questionnaire, including age, gender, blood pressure, height, weight, familial history of hypertension, and previous medical taken/treatment history and calculate the body mass index (BMI) value of all the subjects.

### Inclusion Criteria for Case and Control Group

According to the Chinese hypertension prevention and treatment in 2010, we chose the case group based on the following inclusion criteria: average systolic pressure ≥ 140 mmHg and (or) average diastolic pressure ≥ 90 mmHg; meanwhile, the patients should be without secondary hypertension, coronary heart disease, diabetes, stroke, serious liver, and kidney disorder or any types of malignant tumors. The inclusion criteria of the control group should obey the following rules: healthy individuals with average systolic pressure < 140 mmHg and average diastolic pressure < 90 mmHg; those people who had a history of taking hypertensive drugs should be excluded.

### Selection of SNP Sites

We chose four SNPs on *ADD1* (rs12503220, rs57526673, rs2239728, and rs4961) and three SNPs on *CYP11B2* (rs3802230, rs6433, and rs11781082); some SNPs (like rs4961 and rs3802230) were studied before to find out some relationship with EH with controversial results. SNP selection is based on their allele frequencies and location. Hapmap public database (dbSNP; http://www.ncbi.nlm.nih.gov/SNP/,HAPMAP; http://www.Hapmap.org/index.html.en) was used to identify the disease relevance. In this study, the selected condition is the minor allele frequency (MAF) > 5% in the HapMap Chinese Han Beijing (CHB) population.

### DNA Extraction, Concentration, and Purity Determination

A purification kit (TIANGEN, China) was used to extract human DNA from whole blood samples. The human blood sample was briefly transferred to EP tubes and subsequently mixed with erythrocyte lysate. After being inverted and put at 24°C room temperature for 5 min, the supernatant was removed before adding buffer GA to the EP tube. Proteinase K solution was then dropped into the EP tube and mixed with buffer GB. The mixed complex was then put in a 70°C water bath for 30 min; meanwhile, the EP tube was inverted and mixed several times during the incubating time. Then, 100% ethanol was added to the tube and briefly centrifuged to remove water droplets. The solution we got was subsequently transferred to an adsorption column CB3 and centrifuged at 12000 rpm; the waste was removed, and buffer GD was added to the adsorption column before centrifuging at 12000 rpm again. The rinse solution PW was added to the adsorption column CB3 and centrifuged twice at 12000 rpm before removing the water. The adsorption column was then placed at room temperature for drying. The adsorption column CB3 was finally transferred into a new EP tube and dropped elution buffer TE into the middle part of the membrane. The EP tube was placed at room temperature and then centrifuged at 12000 rpm before collecting the solution into a clean centrifuge tube. NanoDrop 2000C spectrophotometer was used to detect the concentration and purity of the extracted DNA samples. All the extractive aliquots were stored at −20°C.

### PCR Reaction and Primer Design

Primer three online 0.4.0 Software (http://frodo.wi.mit.edu/) was used to design the primer. Reaction buffer, Mg^2+^, dNTP, Taq enzyme, ddH_2_O and primer mix, MJ PTC-200, and Gene Amp PCR detection machine (Norwalk, CT. 06859 United States) were used in the PCR reaction system. The multiplex PCR amplification process includes the following:

1) a predenaturation at 95°C for 2 min; 2) a denaturation at 94°C for 30 s, annealing at 53°C for 90 s, and a start extension at 65°C for 30 s with a total of 40 cycles; and 3) a long extension at 65°C for 10 min. The PCR products were detected using a 3% agarose gel electrophoresis detection system.

### Genotyping

The first-generation sequencing technique multiple ligase detection reaction (LDR) was used in the present study to identify the genotypes of the selected SNPs. The LDR technique aims to identify gene polymorphisms by high-temperature ligase. At the junction site of DNA and two complementary oligonucleotides, the ligation reaction will be terminated once the high-temperature ligase detects a base mismatch of a mutation type. The LDR-specific ligation reaction can be repeated by temperature-controlled cycles to achieve the effect of linear amplification. Spectrometry was used to measure the concentration of DNA (DU530 UV/VIS spectrophotometer, Beckman Instruments, Fullerton, CA, United States). LDR was used to amplify and detect the genotyping reaction, and the genotypes were identified using the GeneMapper 3.0 Software.

### Statistical Analysis

SPSS 17.0, SHEsis, and Excel Software were used to calculate the outcomes in our study, a two-sided *p* <0.05 was considered to be statistically significant. The genotype of all the SNPs was calculated in this case–control study by using χ^2^ tests. Among the control group, the Hardy–Weinberg equilibrium (HWE) was used to identify the frequency of genotype. To keep the reliability of the results and also assess the association between SNPs with the risk of EH, we used unconditional logistic regression analysis with adjustment for gender to identify the constructed 95% confidence intervals (95% CI) and odds ratios (ORs) (Bland and Altman, 2000). In addition, we adjusted gender in the genetic models, together with a linkage disequilibrium structure checked using Haploview 4.2 software.

## Results

### Relationship Between Some General Clinical Data and EH

Comparison of general clinical characteristics among EH patients and the control subgroup were calculated, including triglycerides (TG), total serum cholesterol (TC), high-density lipoprotein (HDL), and low-density lipoprotein (LDL). We extracted these elements from human blood samples and obtained the data by using a biochemical examination. Clinical profiles of all the subjects, including age, height, weight, blood pressure (BP), and BMI were collected and presented in Table 1. The layout of associations between patients’ clinical characteristics with EH was clearly shown in Table 1 and 2. As a result, we identified the average age, BMI, TC, TG, and LDL were significantly associated with EH among Han and Mongolian people (*p <* 0.05); and HDL levels showed a huge upregulation in EH patients when compared with the control group in Mongolian population (*p* < 0.05); no statistical significance was found among the Han population.

**TABLE 1:** Basic clinical characteristics of EH cases and controls in the Han population.

Group	N	Age	SBP (mmHg)	DBP (mmHg)	BMI (kg/M^2^)	TC (mmol/L)	TG (mmol/L)	HDL (mmol/L)	LDL (mmol/L)
Case	198	53.7 ± 12.3	154.7 ± 16.0	91.4 ± 10.4	26.40 ± 3.27	4.97 ± 1.01	1.95 ± 1.13	1.24 ± 0.32	3.12 ± 0.79
Control	212	42.9 ± 12.0	120.0 ± 13.3	74.8 ± 9.0	24.46 ± 3.51	4.63 ± 0.87	1.60 ± 1.01	1.29 ± 0.30	2.85 ± 0.69
t value	—	8.97	23.82	17.28	5.77	3.76	3.32	−1.59	3.64
*p* value	—	<0.01	<0.01	<0.01	<0.01	<0.01	<0.01	NS	<0.01
Male case	112	50.6 ± 13.6	153.7 ± 16.3	92.7 ± 11.0	26.67 ± 3.30	4.82 ± 0.92	1.95 ± 1.05	1.18 ± 0.29	3.07 ± 0.76
Male control	121	42.3 ± 11.7	123.0 ± 10.3	76.7 ± 8.3	25.18 ± 3.19	4.68 ± 0.92	1.86 ± 1.10	1.17 ± 0.26	2.91 ± 0.73
t value	—	5.07	17.03	12.54	3.51	1.18	0.63	0.29	1.63
*p* value	—	<0.01	<0.01	<0.01	<0.01	NS	NS	NS	NS
Female case	86	57.6 ± 9.1	156.0 ± 15.6	89.6 ± 9.2	26.04 ± 3.21	5.17 ± 1.08	1.95 ± 1.22	1.31 ± 0.34	3.18 ± 0.82
Female control	91	42.8 ± 12.4	116.0 ± 15.6	72.3 ± 9.3	23.51 ± 3.70	4.55 ± 0.80	1.25 ± 0.75	1.44 ± 0.29	2.77 ± 0.64
t value	—	8.49	17.06	12.46	4.88	4.34	4.56	−2.73	3.67
*p* value	—	<0.01	<0.01	<0.01	<0.01	<0.01	<0.01	<0.01	<0.01

*p values were calculated by Welch’s t-tests, a *p* value of <0.05 means statistical significance, whereas NS means no significance; BMI, body mass index; TG, triglycerides; TC, total serum cholesterol; HDL, high-density lipoprotein; LDL, low-density lipoprotein.

**TABLE 2:** Basic clinical characteristics of EH cases and controls in Mongolian population.

Group	N	Age	SBP (mmHg)	DBP (mmHg)	BMI (kg/M^2^)	TC (mmol/L)	TG (mmol/L)	HDL (mmol/L)	LDL (mmol/L)
Case	208	56.4 ± 13.5	155.9 ± 14.0	92.3 ± 10.5	27.73 ± 3.97	4.95 ± 0.83	2.02 ± 1.33	1.25 ± 0.33	3.21 ± 0.73
Control	215	44.5 ± 14.1	120.5 ± 12.3	73.4 ± 8.6	25.12 ± 4.44	4.72 ± 1.36	1.52 ± 1.20	1.31 ± 0.32	2.94 ± 0.70
*t* value	—	8.42	27.65	20.24	6.39	2.08	4.07	−2.15	3.93
*p* value	—	<0.01	<0.01	<0.01	<0.01	0.04	<0.01	0.03	<0.01
Male case	139	56.5 ± 13.8	156.3 ± 14.6	93.9 ± 10.0	28.21 ± 3.89	4.90 ± 0.80	2.20 ± 1.50	1.17 ± 0.29	3.16 ± 0.69
Male control	113	44.1 ± 15.4	123.6 ± 11.1	75.7 ± 8.2	25.60 ± 4.93	4.83 ± 1.68	1.77 ± 1.49	1.21 ± 0.29	2.98 ± 0.68
*t* value	—	6.27	20.08	15.57	4.7	0.42	2.24	1.31	2.16
*p* value	—	<0.01	<0.01	<0.01	<0.01	NS	0.03	NS	0.03
Female case	69	56.2 ± 13.2	155.0 ± 12.6	89.1 ± 10.9	26.78 ± 3.98	5.07 ± 0.89	1.66 ± 0.80	1.41 ± 0.35	3.30 ± 0.78
Female control	102	44.9 ± 12.7	117.0 ± 12.6	70.9 ± 8.5	24.59 ± 3.77	4.61 ± 0.88	1.23 ± 0.65	1.43 ± 0.32	2.89 ± 0.73
*t* value	—	5.51	19.33	11.75	3.65	3.34	3.79	−0.33	3.49
*p* value	—	<0.01	<0.01	<0.01	<0.01	<0.01	<0.01	NS	<0.01

*p values were calculated using Welch’s t-tests, a *p* value of <0.05 means statistical significance, whereas NS means no significance; BMI, body mass index; TG, triglycerides; TC, total serum cholesterol; HDL, high-density lipoprotein; LDL, low-density lipoprotein.

### Association Between the SNPs of CYP11B2 and ADD1 With the Risk of EH

We selected four SNPs in *ADD1* and three SNPs in *CYP11B2*. Basic information on these SNPs, including localization, alleles, and bands are shown in Table 3. The minor allele frequency (MAF), ORs, and 95% confidence intervals (95% CI) are shown in Tables 4-7. All the *p* values were calculated using the χ^2^ test to present the comparison between EH patients and healthy control participants. Our finding reported that in the allelic model, *ADD1* rs2239728-A was identified with a 0.74-fold lower risk (95% CI = 0.56–0.98, *p* = 0.033) than rs2293728-C in EH patients when compared to healthy subjects among Han population. Moreover, the *ADD1* rs4961-T demonstrated a 1.37-fold higher risk (95% CI = 1.04–1.81, *p* = 0.027) than allele G in EH patients when compared with healthy controls among Han population. However, no significant difference was found among the Mongolian subjects.

**TABLE 3:** Basic information of seven SNPs.

Gene	SNP ID	Chromosome	Position	Alleles
CYP11B2	rs3802230	8	142911448	A/C
CYP11B2	rs6433	8	142912224	A/G
CYP11B2	rs11781082	8	142918485	A/G
ADD1	rs12503220	4	2848415	A/G
ADD1	rs57526673	4	2878196	C/T
ADD1	rs2239728	4	2904726	A/C
ADD1	rs4961	4	2904980	G/T

**TABLE 4:** Genotype and allele frequency of *CYP11B2* polymorphism between EH and healthy control patients and their relationship with EH risk in the Han population

SNP ID	Genotype Frequency (%)			HWE-*P*	*P* ^*a*^	Alleles Frequency (%)		OR (95%CI)	*P* ^*b*^
rs3802230	AA	AC	CC	—	—	A	C	—	—
Case	27(13.7)	93(47.2)	77(39.1)	0.90	—	147(37.3)	247(62.7)	—	—
Control	30(14.4)	91(43.5)	88(42.1)	0.41	0.756	151(36.1)	267(63.9)	1.05(0.79,1.40)	0.726
rs6433	AA	AG	GG	—	—	A	G	—	—
Case	132(67.0)	62(31.5)	3(1.5)	0.69	—	326(82.7)	68(17.3)	—	—
Control	145(69.4)	58(27.8)	6(2.9)	0.95	0.515	348(83.3)	70(16.7)	0.96(0.67,1.39)	0.846
rs11781082	AA	AG	GG	—	—	A	G	—	—
Case	2(1.0)	36(18.3)	159(80.7)	0.98	—	40(10.2)	354(89.8)	—	—
Control	2(1.0)	41(19.6)	166(79.4)	0.76	0.925	45(10.8)	373(89.2)	0.94(0.60.1.47)	0.775

SNP, single nucleotide polymorphism; MAF, minor allele frequency; HWE, Hardy–Weinberg equilibrium; ORs, odds ratios; CI, confidence interval; *p* were adjusted by gender and age; *P* ^*a*^ presents the difference of genotypes between EH and healthy control patients; *P* ^*b*^ shows the comparison of alleles between EH and healthy control subgroup; a *p* value of <0.05 means statistical significance.

**TABLE 5:** Genotype and allele frequency of *CYP11B2* polymorphism between EH and healthy control patients and their relationship with EH risk in the Mongolian population.

SNP ID	Genotype frequency (%)			HWE-*P*	*P* ^*a*^	Alleles frequency (%)		Or (95%CI)	*P* ^*b*^
rs3802230	AA	AC	CC	—	—	A	C	—	—
Case	31 (15.5)	93 (46.5)	76 (38.0)	0.77	—	155 (38.8)	245 (61.3)	—	—
Control	35 (16.6)	94 (44.5)	82 (38.9)	0.36	0.913	164 (38.9)	258 (61.1)	1.00 (0.75.1.32)	0.974
rs6433	AA	AG	GG	—	—	A	G	—	—
Case	145 (72.5)	48 (24.0)	7 (3.5)	0.24	—	338 (84.5)	62 (15.5)	—	—
Control	148 (70.1)	57 (27.0)	6 (2.8)	0.86	0.746	353 (83.6)	69 (16.4)	1.07 (0.73.1.55)	0.739
rs11781082	AA	AG	GG	—	—	A	G	—	—
Case	2 (1.0)	37 (18.5)	161 (80.5)	0.94	—	41 (10.3)	359 (89.8)	—	—
Control	2 (0.9)	41 (19.4)	168 (79.6)	0.77	0.962	45 (10.7)	377 (89.3)	0.96 (0.61.1.50)	0.846

SNP, single nucleotide polymorphism; MAF, minor allele frequency; HWE, Hardy–Weinberg equilibrium; ORs, odds ratios; CI, confidence interval; *p* were adjusted by gender and age, *P* ^*a*^ presents the difference of genotypes between EH and healthy control patients; *P* ^*b*^ shows the comparison of alleles between EH and healthy control subgroup; a *p* value of <0.05 means statistical significance.

**TABLE 6:** Genotypes and allele frequency of *ADD1* polymorphism between EH and healthy control patients and their relationship with EH risk in the Han population.

SNP ID	Genotype frequency (%)			HWE-*P*	*P* ^*a*^	Alleles frequency (%)		Or (95%CI)	*P* ^*b*^
rs12503220	AA	AG	GG	—	—	A	G	—	—
Case	4 (2.1)	42 (21.6)	148 (76.3)	0.62	—	50 (12.9)	338 (87.1)	—	—
Control	8 (3.9)	53 (25.6)	146 (70.5)	0.26	0.333	69 (16.7)	345 (83.3)	0.74 (0.50.1.10)	0.132
rs57526673	CC	CT	TT	—	—	C	T	—	—
Case	125 (64.4)	61 (31.4)	8 (4.1)	0.87	—	311 (80.2)	77 (19.8)	—	—
Control	138 (66.7)	57 (27.5)	12 (5.8)	0.07	0.560	333 (80.4)	81 (19.6)	0.98 (0.69.1.39)	0.921
rs2239728	AA	AC	CC	—	—	A	C	—	—
Case	37 (19.1)	88 (45.4)	69 (35.6)	0.35	—	162 (41.8)	226 (58.2)	—	—
Control	48 (23.2)	108 (52.2)	51 (24.6)	0.53	0.056	204 (49.3)	210 (50.7)	0.74 (0.56.0.98)	0.033*
rs4961	TT	GT	GG	—	—	T	G	—	—
Case	69 (35.6)	88 (45.4)	37 (19.1)	0.35	—	226 (58.2)	162 (41.8)	—	—
Control	51 (24.6)	107 (51.7)	49 (23.7)	0.63	0.055	209 (50.5)	205 (49.5)	1.37 (1.04.1.81)	0.027*

SNP, single nucleotide polymorphism; MAF, minor allele frequency; HWE, Hardy–Weinberg equilibrium; ORs, odds ratios; CI, confidence interval; *p* values were adjusted using gender and age, *P* ^*a*^ presents the difference of genotypes between EH and healthy control patients, *P* ^*b*^ shows the comparison of alleles between EH and healthy control subgroup, a*p* value of <0.05 was regarded as statistical significance. All the bold values mean their p value < 0.05, presenting a statistical significance.

**TABLE 7:** Genotypes and allele frequency of *ADD1* polymorphism between EH and healthy control patients and their association with EH risk in the Mongolian population

SNP ID	Genotype frequency (%)			HWE-*P*	*P* ^*a*^	Alleles frequency (%)		Or (95%CI)	*P* ^*b*^
rs12503220	AA	AG	GG	—	—	A	G	—	—
Case	3 (1.5)	49 (24.3)	150 (74.3)	0.66	—	55 (13.6)	349 (86.4)	—	—
Control	2 (1.0)	36 (17.5)	168 (81.6)	0.96	0.186	40 (9.7)	372 (90.3)	1.47 (0.95.2.26)	0.082
rs57526673	CC	CT	TT	—	—	C	T	—	—
Case	144 (71.3)	49 (24.3)	9 (4.5)	0.08	—	337 (83.4)	67 (16.6)	—	—
Control	146 (70.9)	55 (26.7)	5 (2.4)	0.90	0.481	347 (84.2)	65 (15.8)	0.94 (0.65.1.37)	0.754
rs2239728	AA	AC	CC	—	—	A	C	—	—
Case	44 (21.8)	95 (47.0)	63 (31.2)	0.47	—	183 (45.3)	221 (54.7)	—	—
Control	30 (14.6)	105 (51.0)	71 (34.5)	0.38	0.166	165 (40.0)	247 (60.0)	1.24 (0.94.1.64)	0.130
rs4961	TT	GT	GG	—	—	T	G	—	—
Case	63 (31.2)	95 (47.0)	44 (21.8)	0.47	—	221 (54.7)	183 (45.3)	—	—
Control	71 (34.5)	105 (51.0)	30 (14.6)	0.38	0.166	247 (60.0)	165 (40.0)	0.81 (0.61.1.07)	0.130

SNP, single nucleotide polymorphism; MAF, minor allele frequency; HWE, Hardy–Weinberg equilibrium; ORs, odds ratios; CI, confidence interval; *p* values were adjusted using gender and age; *P* ^*a*^ presents the difference of genotypes between EH and healthy control patients; *P* ^*b*^ shows the comparison of alleles between EH and healthy control subgroup; a *p* value of <0.05 means statistical significance.

After the identification that alleles frequency distributions of rs4961 and rs2239728 in *ADD1* were correlated to EH risk in the Han population when compared with healthy subjects, our step-by-step exploration continued to adjust these SNPs under a dominant and recessive genetic model. An unconditional logistic regression was used to identify the accurate association between these detected SNPs and the risk of EH. The dominant genetic model shows that rs2239728-A carrier (AA + AC) was 0.59-fold (95%CI: 0.39–0.91) lower than CC carrier in EH patients than in healthy controls. In addition, rs4961-G carrier (GG + GT) presents a 0.59-fold (95%CI: 0.39–0.91) lower than TT carrier in EH patients than in healthy subjects (Table 8).

**TABLE 8:** Association between *ADD1* SNPs with EH risk in the Han population (based on logistical tests).—

>SNP ID	Dominant	*p*	OR (95%CI)	Recessive	*p*	OR (95%CI)
rs2239728	AA + AC/CC	—	—	AA/AC + CC	—	—
Case	125/69	—	—	37/157	—	—
Control	156/51	0.017*	0.59 (0.39.0.91)	48/159	0.31	0.78 (0.48.1.26)
rs4961	GG + GT/TT	—	—	GG/GT + TT	—	—
Case	125/69	—	—	37/157	—	—
Control	156/51	0.017*	0.59 (0.39.0.91)	49/158	0.26	0.76 (0.47.1.23)

SNP, single nucleotide polymorphism; ORs, odds ratios; CI, confidence interval; AIC, Akaike’s information criterion; BIC, Bayesian information criterion. *p* values were calculated with logistic analysis. *p < 0.05, statistical significance. All the bold values mean their p value < 0.05, presenting a statistical significance.

### Adjustment Based on Gender

Previous findings indicated the rates of males or female suffering from EH was different, we subsequently adjusted the two significantly positive SNPs (rs2239728, rs4961) based on gender. Interestingly, we found a statistical difference between males and females (Table 9). Furthermore, no SNPs showed any difference among males. However, patients with rs2239728-A presented a 0.63-fold risk (95%CI: 0.41–0.96) than those with –C in EH patients when compared with healthy controls. Furthermore, the rs4961-T carrier was associated with a 1.63-times higher risk (95%CI: 1.06–2.49) than –G in females in EH subjects when compared with healthy participants.

**TABLE 9:** Association between ADD1 polymorphisms and the risk of EH among the Chinese Han population (adjusted by gender).

SNP ID	Genotype			HWE-*P*	*P* ^*a*^	Allele		OR (95%CI)	*P* ^*b*^
rs2239728	AA	AC	CC	—	—	A	C	—	—
Male case	26	46	38	0.11	—	98	122	—	—
Male control	28	59	30	0.92	0.300	115	119	0.83 (0.58.1.20)	0.326
Female case	11	42	31	0.58	—	64	104	—	—
Female control	20	49	21	0.40	0.087	89	91	0.63 (0.41.0.96)	0.033*
rs4961	TT	GT	GG	—	—	T	G	—	—
Male case	38	46	26	0.11	—	122	98	—	—
Male control	30	59	28	0.92	0.300	119	115	1.20 (0.83.1.74)	0.326
Female case	31	42	11	0.58	—	104	64	—	—
Female control	21	48	21	0.53	0.073	90	90	1.63 (1.06.2.49)	0.025*

ORs: odds ratios; CI: confidence interval; AIC, Akaike’s information criterion.

BIC: Bayesian information criterion. *p* values were adjusted based on gender.

*P* ^*a*^ presents the difference of genotypes between EH and healthy control patients, whereas *P* ^*b*^ shows the comparison of alleles between EH and healthy control subgroup, a *p* value of <0.05 was regarded as statistically significant. All the bold values mean their p value < 0.05, presenting a statistical significance.

### Association Between Haplotype of CYP11B2 and ADD1 With EH Risk

**FIGURE 1:**
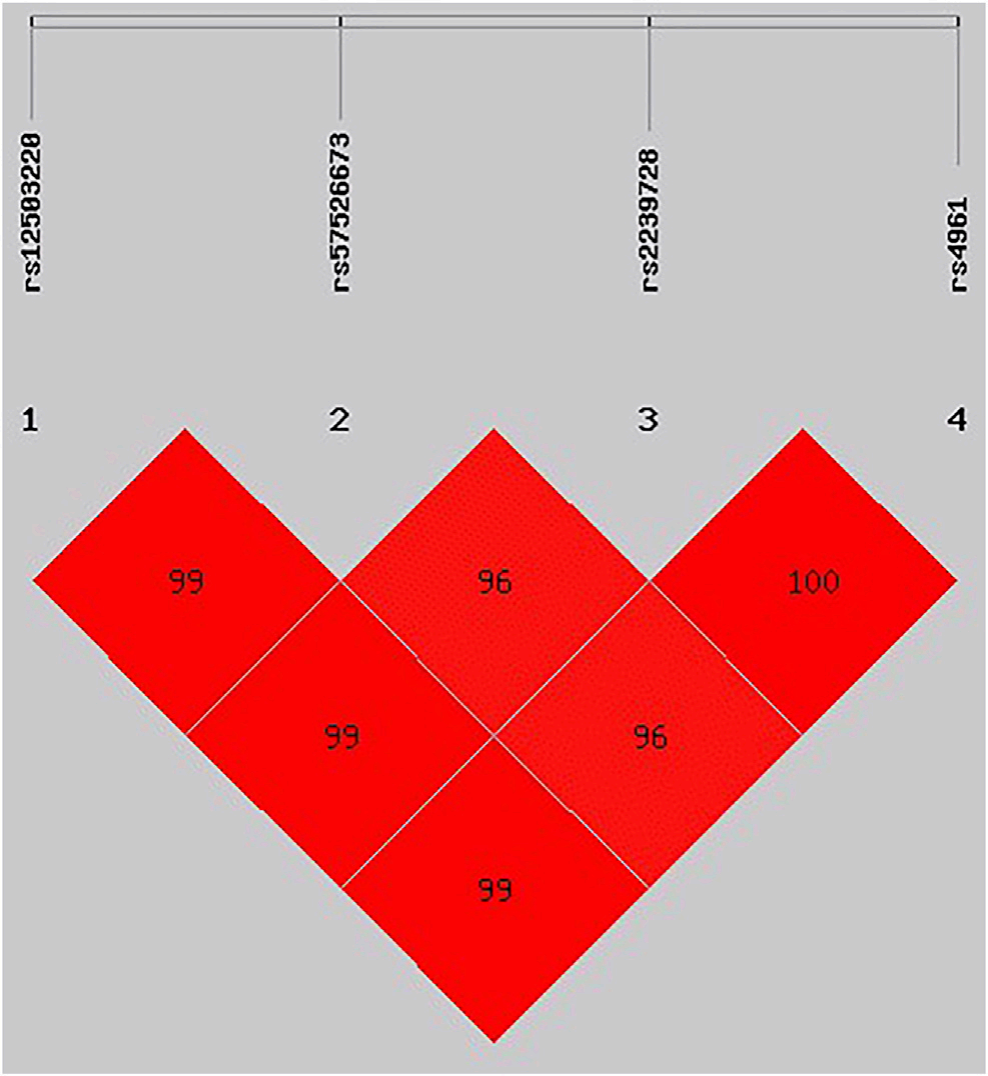
We used the parameters r2 and D′’ to analyze the linkage disequilibrium (LD) of the SNPs on ADD1. Significant LD is indicated by bright red standard colors.

In our study, we used SHEsis Software to analyze linkage disequilibrium (LD) and then identify the association between the haplotypes of *CYP11B2* and *ADD1* with EH risk by using χ^2^ and logistic tests. We finally found that Grs12503220 Crs57526673 Crs2239728 Trs4961 haplotype was associated with an increased risk of EH in the Han population (OR: 1.36, 95% CI: 1.03–1.80, *p* = 0.029). We did not find any relationship between the haplotype of *CBY11B2* and EH risk in either population (Table 10 and Figure 1).

**TABLE 10:** Haplotype frequencies of *ADD1* polymorphisms and EH risk in Han population.

Haplotype	Case (%)	Control (%)	*p*	OR (95%CI)
ACAG	50.00 (0.129)	69.00 (0.167)	0.129	0.74 (0.50.1.09)
GCAG	36.09 (0.093)	55.16 (0.133)	0.071	0.67 (0.43.1.04)
GCCT	224.91 (0.580)	207.84 (0.502)	0.029*	1.36 (1.03.1.80)
GTAG	75.91 (0.196)	79.85 (0.193)	0.934	1.02 (0.72.1.44)

Global haplotype association *p* value: 0.19; ORs, odds ratios; CI, confidence interval; *p < 0.05, statistical significance.All the bold values mean their p value < 0.05, presenting a statistical significance.

## Discussion

Genetic identification can be used as early screening control and provide guidance for a series of diseases, such as hypertension. Getting a better understanding and insight into the relationship between genes and EH is helpful to predict EH development and improve protection as well. Previous studies have investigated that *CYP11B2* and *ADD1* are related to the risk of EH, but the results are controversial in different races and areas (Morrison et al., 2001; Morrison et al., 2002; Gong et al., 2009; Niu et al., 2010; Ji et al., 2013). However, insights into the association between both genes and EH in Inner Mongolia province are lagging behind. Our study is the first to investigate the association between genetic variants of *ADD1* and *CYP11B2* with the risk of EH among the Han and Mongolian populations in Inner Mongolia province by using a case–control study. Our present findings immediately reported two SNPs in *ADD1* are related to EH risk among females in the Han population, demonstrating that allele A of rs2239728 is a protective factor of EH, whereas allele T of rs4961 is a risky factor. Interestingly, our finding is a novel extension, reporting that the genetic polymorphisms of *ADD1* present both protective and risky relationships with EH but also demonstrate a gender difference among the Chinese Han population. Moreover, these findings not only strengthen the importance of ADD1 in EH prognosis but also highlight novel SNPs with high LD, which can be a great tool in EH risk prediction and further target detection.

Adducin is a type of erythrocyte cytoskeleton protein that is composed of three kinds of subunits (α, β, and γ). Adducin can bind to the hemoglobulin-actin complex to improve the binding of hemoglobulin. In general, adducin participates in the membrane–ion transport, the process of cell junction, and signal transduction, especially playing a key role in the sodium pump working and various Na + transport mechanisms. *ADD1*, which is the coding gene of α-adducin, was found to have a mutation in the site of the 10th exon 614th basic group, which means that the 460 Gl y (G) is replaced by 460Trp (T). The change of G to T can influence the stability of adducin structure and can further change the assembly of actin cytoskeleton and the activity of the sodium pump, causing increasing reabsorption of Na+ by renal tubular epithelial cells, finally resulting in water and sodium retention, ultimately leading to EH. Studies of Gly460Trp (rs4961) genetic polymorphism on subjects from different nations and areas presented some differences in China. An investigation made by Ju and his assistants has shown that rs4961 is related to EH in the northern area of China (Ju et al., 2003). Liu also found that Gly460Trp (rs4961) polymorphism is associated with an increased risk of EH in the Chinese population (Liu et al., 2011). Both studies are consistent with our outcome about *ADD1* (rs4961). At the molecular level, one previous research showed that *ADD1*-T allele carriers presented an increased level of renal tubular sodium reabsorption and retention (Manunta et al., 1998a) because its renal pressure–natriuresis slop is declined. Manunta et al. investigated the mechanism in detail, that is, the genetic mutation of G to T regulates sodium transport by regulating the biochemical activity of sodium–potassium ATPase or by modifying the assembly of actin cytoskeleton (Manunta et al., 1998b). A following overall review in 2010 showed that there exists a statistically significant association between the Gly460 T allele and salt sensitivity among the Asian population (Wang et al., 2010). Here the previous findings and molecular mechanism are consistent with our results since all the subjects we chose are living in Inner Mongolia, which is one of the largest salt-consuming regions in China. Our study also found that the rs2239728 A allele is a protective factor. Although we have not found any articles about this SNP and EH, one previous study about ion homeostasis in Ménière’s Disease showed that mutation of rs2239728 is associated with Na+ exchange at a cellular level (Teggi et al., 2017). As a result, a change of C to A of rs2239728 might have some positive effects on adducin structure and consolidate the assembly of actin cytosketon, further decreasing the water and sodium retention and then ultimately reducing the risk of EH.

According to the HWE detection, we can see that four SNPs in *ADD1* all conform to HWE among the case and control groups (*p* > 0.05), which indicates that the sample collection method is a reliable and genetic frequency that can present the genetic distribution of the population. After adjusting for gender, we found that the distribution of rs2239728 and rs4961 genotypes in both groups still conform to HWE. The outcome showed that both SNPs were only significant among females in the Han population. The risk of EH in females with rs4961-T carriers was 1.63-fold higher than G allele carriers, whereas rs2239728-A allele carriers were associated with 0.63-fold than that of C allele carriers. The results identified that gender is a confounding factor in influencing the risk of EH. Together, this kind of information can be used as genetic targets for EH predicting and therapy.

In addition, Chu et al. (2014) and Cheng et al. (2011) reported that haplotypes might be a piece of better evidence than a single SNP for affecting clinical reaction. Our study is the first to investigate haplotypes among *ADD1* rs12503220, rs57526673, rs2239728, and rs4961. The haplotype analysis showed that the Grs12503220 Crs57526673 Crs2239728 Trs4961 haplotype was related to an increased risk of EH in the Han population. Further studies are needed to find out more insightful knowledge of the mechanism.

## Conclusion

Our present study investigated the relationship between genetic variants of *ADD1* and *CYP11B2* with the risk of EH in the Han and Mongolian populations in Inner Mongolia province through a case–control study. Our findings identified two SNPs in *ADD1* that were related to EH risk among females in the Han population, as allele A of rs2239728 was a protective factor of EH, whereas allele T of rs4961 was a risky factor. However, genetic polymorphisms of *CYP11B2* show no significant association in the Mongolian population.

## Data Availability

The data presented in the study are deposited in the Figshare repository (https://figshare.com/), accession number https://doi.org/10.6084/m9.figshare.20016170.v1 https://doi.org/10.6084/m9.figshare.20025680.v1, and https://doi.org/10.6084/m9.figshare.20025503.v1
