# Supplementary material for: ADD1 Single Nucleotide Polymorphisms Are Associated With Essential Hypertension Among Han and Mongolian Population in Inner Mongolia Area
Source: Front Genet. 2022 Aug 12;13:931803. doi: 10.3389/fgene.2022.931803 (PMC9412030; doi:10.3389/fgene.2022.931803)
Supplement: Supplementary file 3 [file DataSheet1.PDF]

[illegible]

内蒙古医科大学科学技术处文件

编号(№)：YKD2015107

内蒙古医科大学生物医学科研伦理审批件

我校常培叶申请的国家自然科学基金项目《多巴胺转运体基因多态性与帕金森病易感性及其功能影像学的相关研究》经过医学伦理委员会的审核，符合伦理原则，同意申报国家自然科学基金项目。

内蒙古医科大学医学伦理委员会

伦理委员会主任委员签章 牛广明印

2015 年 3 月 11 日
